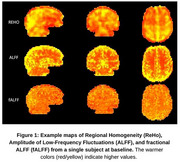# Reproducible Neuroimaging Analysis Pipelines for Dementia Research in Resource‐Limited Settings: Experience with fMRI Processing and Analysis

**DOI:** 10.1002/alz70856_104116

**Published:** 2025-12-24

**Authors:** Philip Nkwam, Ethan Draper, Jasmine Cakmak, Alfonso Fajardo, Kesavi Kanagasabai, Channelle Tham, Oluwateniola Akinwale, Charity Umoren, Olusola Aremu, Nsiah Donkor Anita, Confidence Raymond, Farouk Dako, Udunna Anazodo, Abdalla Z Mohamed

**Affiliations:** ^1^ University of Lagos, Lagos, Nigeria; ^2^ Montreal Neurological Institute, Montreal, QC, Canada; ^3^ Montreal Neurological Institute, McGill University, Montreal, QC, Canada; ^4^ Integrated Program in Neurosciences, McGill University, Montréal, QC, Canada; ^5^ Department of Medical Biophysics, Western University, London, ON, Canada; ^6^ Radboud University, Nijmegen, Netherlands; ^7^ Johns Hopkins University, Baltimore, MD, USA; ^8^ Medical Artificial Intelligence Laboratory, Crestview Radiology Ltd, Lagos, Nigeria; ^9^ Department of Medicine, Lagos State University, Lagos, Nigeria; ^10^ Department Of Medical Imaging Technology, University For Development Studies, Tamale, Ghana; ^11^ Department of Biomedical Engineering, McGill University, Montreal, QC, Canada; ^12^ Perelman School of Medicine, University of Pennsylvania, Philadelphia, PA, USA; ^13^ Medical Artificial Intelligence (MAI) Laboratory, Crestview Radiology Limited, Lagos, Nigeria; ^14^ Center for Brain and Health, New York University‐ Abu Dhabi, Abu Dhabi, United Arab Emirates

## Abstract

**Background:**

Resting‐state functional MRI (rs‐fMRI) allows us to investigate disruptions in brain connectivity associated with Alzheimer's disease (AD) progression. However, researchers in Low‐ and Middle‐Income Countries (LMICs) face significant barriers in analyzing fMRI data due to limited computational resources, and lack of standardized preprocessing pipelines tailored towards limited resource environments. We created a reproducible, resource‐efficient cloud‐based rs‐fMRI analysis pipeline specifically designed from open‐source preprocessing tools to address these challenges and empower LMIC researchers to conduct comparable neuroimaging research.

**Method:**

This work was performed as part of the CONNExIN (COmprehensive Neuroimaging aNalysis Experience In resource‐constraiNed Settings) Program, a neuroimage analysis training program for African neuroscience researchers. A team of CONNExIN students utilized the Pre‐symptomatic Evaluation of Novel or Experimental Treatments for Alzheimer's Disease (PREVENT‐AD) dataset to develop an open‐access analysis pipeline. The dataset included 75 subjects, each with high‐resolution anatomical T1‐weighted images and rsfMRI datasets acquired at baseline and four annual follow‐ups (12‐ 48 months). Our approach used Neurodesk (**1**) running on Google Colab to process and analyze the data using Google Cloud Engine instance (Intel(R) Xeon(R) CPU 3.10 GHz and 32GB RAM) running on a standard internet connection. Standard fMRI preprocessing steps (slice timing correction, motion correction, skull stripping and spatial normalization) were implemented to process the data. Functional connectivity metrics (Amplitude of Low‐Frequency Fluctuations (ALFF), fractional ALFF (fALFF), and Regional Homogeneity (ReHo)) were estimated on a subset of the data to evaluate the functionality of our approach.

**Result:**

Preliminary analysis on data from 17 subjects taking around 20 minutes/timepoint/subject to generate reliable ALFF, fALFF, and ReHo maps (Figure 1), across all timepoints). Documentation of the full analysis pipeline will be made available on Protocol.io, ensuring transparency, version control, and facilitating reproducibility for LMIC researchers.

**Conclusion:**

We introduced a cloud‐based low‐resource rs‐fMRI analysis pipeline to address computational constraints in LMIC research settings. Our approach will be applied to the whole PREVENT‐AD dataset and an ongoing African dementia study. A reliability analysis across LMIC groups, running the same pipeline on the same dataset, will assess its generalizability for resource‐efficient rs‐fMRI analysis.